# Regenerative Medicine of Liver: Promises, Advances and Challenges

**DOI:** 10.3390/biomimetics6040062

**Published:** 2021-10-20

**Authors:** Saiful Ali, Nasira Haque, Zohya Azhar, Morvarid Saeinasab, Farshid Sefat

**Affiliations:** 1 Department of Biomedical and Electronics Engineering, School of Engineering, University of Bradford, Bradford BD7 1DP, UK; saiful_471@hotmail.com (S.A.); haquenasira@gmail.com (N.H.); zohya_05@outlook.com (Z.A.); 2 Department of Biology, Faculty of Science, Ferdowsi University of Mashhad, Mashhad 9177948974, Iran; m.saeinasab@gmail.com; 3 Interdisciplinary Research Centre in Polymer Science & Technology (Polymer IRC), University of Bradford, Bradford BD7 1DP, UK

**Keywords:** liver, scaffold, regenerative medicine, hepatocyte, stem cell

## Abstract

Liver tissue engineering is a rapidly developing field which combines the novel use of liver cells, appropriate biochemical factors, and engineering principles, in order to replace or regenerate damaged liver tissue or the organ. The aim of this review paper is to critically investigate different possible methods to tackle issues related with liver diseases/disorders mainly using regenerative medicine. In this work the various regenerative treatment options are discussed, for improving the prognosis of chronic liver disorders. By reviewing existing literature, it is apparent that the current popular treatment option is liver transplantation, although the breakthroughs of stem cell-based therapy and bioartificial liver technology make them a promising alternative.

## 1. Introduction

The liver is a very integral organ in the human body and in healthy adults’ the weight of this organ is approximately 1.4 kg, which makes it the largest gland in the human body. The rib cage protects the liver; therefore, it is unusual to be able to feel the liver from outside the body [1].

There are many diseases/disorders that exist related to this vital organ which need serious consideration, otherwise the patients face many problems and in severe cases could cause death. A trend of an increasing ageing population can be seen within more economically developed countries (MEDC). It has been identified that age has an adverse effect on the severity of a wide variety of prominent liver disorders, such as non-alcoholic fatty liver disease (NAFLD) [2]. Alongside age, poor lifestyles, and diet, in these countries contribute to the growing number of cases of liver related diseases. End-stage liver diseases can currently only be combatted using liver transplantation (LT); however, recently alternative treatment options and therapies have been explored [3]. This review will further investigate these possibilities.

**Figure 1:**
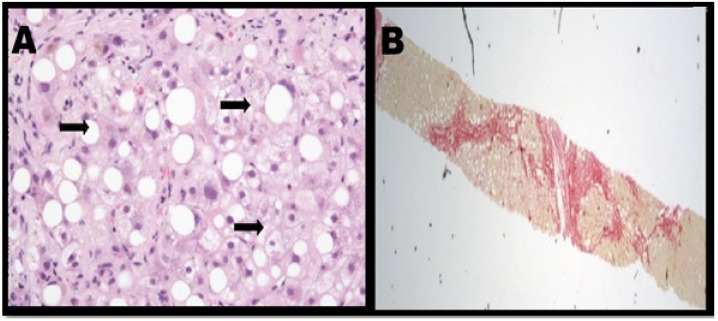
(**A**) Steatohepatitis with notable ballooning of hepatocytes. (**B**) Patient with steatohepatitis has severe fibrosis [4].

Presently, NAFLD is regarded as the most common chronic liver disease in developed countries due to the rise in a growing number of obese patients. The disease is accountable for increasing liver-related mortality, along with contributing to the development of other prominent diseases, such as cardiovascular disease and type 2 diabetes [2]. NAFLD is categorised by excess accretion of fat in hepatocytes, leading to steatosis; a condition that occurs when the fat content in the liver is >5%. Hepatocyte apoptosis occurs in the presence of steatosis, accompanied by portal and lobular inflammation, which results in the manifestation of non-alcoholic steatohepatitis (Figure 1) [4,5]. Cirrhosis may occur with continued collagen accumulation and consequential vascular remodelling. Alcoholic fatty liver disease (AFLD) shares various histological features of NAFLD which include inflammation, steatosis and fibrosis within lobules. However, contrary to the causal exposure to excess alcohol that gives rise to AFLD, patients with NAFLD have been identified to be typically obese, insulin resistant and do not drink excess alcohol [6,7].

Hepatitis is the term given for several viral diseases that initiate inflammation of the liver. The hepatitis C virus (HCV) appears to be the most common form of hepatitis, as the prevalence of this disease may be as high as 5–15% in certain parts of the world. Once HCV enters the hepatocytes by the process of receptor mediated endocytosis and begins multiplication, it causes damage to the hepatocyte. This is mostly achieved through utilisation of the host’s own immune response. Host cells release interferons as an effective defence mechanism against intra-cellular viral infection. However, HCV possesses the ability to avoid this natural interferon-mediated response due to complicated actions of its genomic proteins [8,9,10].

Following initial acute infection, around 45% of patients may have an effective cell-mediated response, leading to the elimination of HCV. However, majority of infected patients are unable to remove the virus, which leads to chronic infection along with liver damage. Initial HCV diagnosis involves the use of serology or using alanine transaminase as a biomarker to assess liver damage [8,11].

Biliary atresia is a very rare and serious disease that affects infants, generally within their first few weeks of birth [12]. The intrahepatic/extrahepatic bile ducts become obstructed and scarred because of this disorder, which means bile can no longer flow to the intestines, hence resulting in eventual liver damage. The pathogenesis of this disease is not yet fully understood. Commonly, infants who are affected by biliary atresia do not have a family history of liver disorders [13]. Symptoms involve persistent jaundice, although the infant can still appear to grow healthily. Early signs include the infant experiencing dark coloured urine, acholic stools, the sclera becoming yellow and haemorrhage. If biliary atresia is found after approximately >90 days, it is likely that progressive hepatic fibrosis and cirrhosis, will cause the infant’s growth to halt and splenomegaly [14,15].

Wilson’s disease is a rare inherited disorder that causes copper imbalance in the human body, which can cause neurological issues and hepatic damage. ATP7B has a vital role in copper metabolism and is the defective gene which must be inherited from both parents, for the disease to be expressed [16]. Excess copper accumulation occurs, within Wilson’s disease sufferers, and in some cases, this can become life threatening. Although Wilson’s disease is present when the affected individual is born, they may not experience symptoms until copper accumulation has occurred in organs such as the liver and the brain [17].

There are numerous types of liver cancer comprising heterogeneous clusters of malignant tumours. These range from hepatocellular carcinoma; the most common type, to hepatocellular cholangiocarcinoma, intrahepatic cholangiocarcinoma and fibrolamellar which are less common [18]. Most causes of liver cancer are either idiopathic or metastatic which is when cancer initiates in a different region of the body, and then spreads to the liver. Cancer occurs due to the mutation of liver cells into pre-cancerous lesions, that progress into malignant tumours. This transformation occurs through the interaction of genetic factors with biological, physical and chemical carcinogens [19]. Common signs and symptoms of liver cancer include upper abdominal pain and swelling, loss of appetite, yellow discoloration of skin and sclera (jaundice) and white chalky stools [20].

Cirrhosis occurs due to persistent, long-term damage of the liver leading to scarring, known as fibrosis. Fibrosis materialises from many liver diseases such as chronic alcoholism and hepatitis; it progresses in relation to the extent of damage caused, host factors and the environment. It leads to the deformation of hepatic vasculature, as the arterial blood supply is propelled into the hepatic outflow, causing perpetual damage [21,22].

## 2. Treatment

For the past decades, many clinicians and researchers tried various methods to tackle issues related to liver and some of these techniques were successful while most of them failed. Liver transplantation is the one with the most acceptable rate of success which will be discussed in the next section. Liver-on-chip, bioartificial liver, various scaffolds and stem cell therapies are other successful techniques but still not fully functional as the research has not been completed yet. In the next sections, the advantage and challenges related to this technique will be discussed.

### 2.1. Liver Transplantation

A liver transplant consists of a surgical procedure that is carried out to remove a non-functional liver to replace it with a healthy donor liver [23]. Early referral increases the chance of LT success, since transplant centres have time to fully review the individual’s health. Additionally, it means that the individual can assess the options available to them without experiencing pressure. It is essential that all other measures to manage the patient’s disease have been exhausted, since there are many risks involved with LTs [24]. Transplantation could be done via different routes such as donation after circulatory death (DCD), brainstem death (DBD) and transplantation from healthy person. DCD which also known as donation after cardiac death, refers to the recovery of organs in the case of transplantation from a person with cardio-respiratory criteria whereas DBD is a death from neurological criteria which also known as brain-stem death or brain death). On the other hand, liver donation from a living donor can be done by donating part of liver, such has a lobe, to a person in need.

The patient must be healthy enough to survive the surgical procedure and must be able to comply with medication regimes and medical advice [24,25]. Burra et al., 2011 found that adherence to medication regimes was directly connected to the success of a LT. Graft loss and rejection can occur if there is non-compliance to anti-rejection medication. Also, it was found that lack of adherence to medical advice such as alcohol/smoking prohibition after the transplant, could be associated with de novo tumours and further health complications. Generally, a trend was displayed that paediatric LT patients had a greater non- adherence compared to adult LT patients. In the literature, there is a varied range of between 3–47% for non-compliance to clinical appointments and a range of 15–40% for the rate of non-compliancy to medication regimes in adults who undergo a LT. The reason behind the large variation in these ranges, is that in medical literature non-compliancy is defined in various ways, and many diverse methods are used to measure adherence.

The criteria for referral for a LT are dependent on the clinical situation. These can be categorised into fields such as acute/chronic liver failure, variant syndromes and hepatocellular carcinoma [24]. Individuals who suffer from liver failure/cancer, where the condition cannot be managed, may opt for this treatment. Acute liver failure occurs rapidly across a short period of time; it is usually caused by medication-induced liver damage. LTs are more commonly used to treat chronic liver failure, which occurs gradually [23]. Adam et al., 2012 reported that the most common indication of LT within Europe (1968–2009) was cirrhosis, this is where liver function becomes impaired due to scarring. Factors that cause liver cirrhosis include AFLD, NAFLD, genetic diseases and hepatitis B/C [26]. Additionally LT may be used as a treatment option for individuals suffering from primary liver cancers [23].

The LT process involves undergoing an assessment in which the patient is questioned about how symptoms affect their quality of life, medical history and if the patient has a history of drug and alcohol abuse. During this assessment period the patient will have tests carried out on them such as blood tests, X-rays, ECG, spirometry and an endoscopy. The LT team will then decide if the patient is suitable for transplantation, and they will be placed onto a waiting list. If there is a low success rate, the LT team may instead decide to monitor symptoms until the patient’s condition changes and a reassessment is conducted [27].

The number of individuals requiring a LT greatly exceeds the availability of deceased donor livers. It is possible to conduct a living donor transplant and combat this issue. This is because after a segment of the liver is surgically removed, the liver can return to its original size, through regeneration [23]. Additionally, split liver transplants and living associated partial donor procedures are some of the surgical options that are conducted to improve the supply of livers available for transplantation [28,29].

During the surgical procedure of a LT, the diseased liver’s blood supply and bile ducts are disconnected. The diseased liver is replaced with the healthy donor liver, then the bile ducts and blood supplying vessels are reconnected onto it [23,27]. Generally, LTs are successful as many people live for up to 20 years after a liver transplant, but it can take at least a year for full recovery after surgery. After a LT, the individual must take immuno-suppressants for the rest of their lives, have a healthy lifestyle and regularly visit the doctor to see the progress of the new liver [27].

There is a substantial probability of complications associated with the LT procedure and the anti-rejection medications that are required after. Therefore, this mode of treatment is only recommended if the benefits of an LT outweigh the large risks. Complications can occur during the surgical procedure with bile duct leaks or shrinkages, rejection of the donor liver and function failure of the donated liver. Long-term effects can include a recurrence of liver disease within the transplanted liver. Side effects from the anti-rejection diseases can include bone thinning, high blood pressure/cholesterol and diabetes. Since anti-rejection medication are immuno-suppressant, the individual may also become more prone to infection [23,27].

Liver transplantation is an effective and primary treatment for hepatic failure/disease, although donor livers are not readily available for all the patients who need a LT. The LT is the only treatment which directly affects mortality. Despite advances in the surgical procedure of an LT, organ allocation developments, liver transplantation alone cannot ever completely meet the growing demand. Thus, regenerative therapies are being investigated to combat the issue of the supply of donor livers, not meeting the high demand. This report further explores the main alternative treatment options in detail.

### 2.2. Liver-on-a-Chip

**Figure 2:**
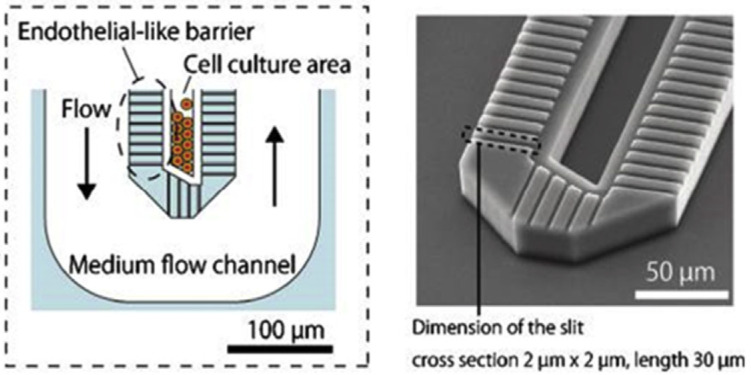
Liver-on-a-chip (LoC) devices have the potential to replicate the hepatic cord architecture [31].

Microfluidic chip-based technology is an advancing platform for cell culture in vitro. The function and complex structures of human organs can be mimicked, to assemble human physiological models, and establish the progression of toxicity research and drug discovery [30]. Organ-on-chips are useful for testing disease models, drug delivery and for further study of tissue/organ level behaviour (Figure 2) [4,31]. Cardiomyopathy and simulations of liver infection by the hepatitis B virus are an example of how organ-on-chip systems can be utilised. However, limitations such as the macro-scale architecture of organ functions like brain cognition and bone mechanical function, is not easily modelled on chips. A complication that hinders the progress of this device as the 3D the liver-on-a-chip (LoC) system becomes more complex, is that it is increasingly challenging to obtain a high-resolution microscopic live image of processes within the liver [32].

The liver is one of the many organs that has been replicated using this progressive chip-based technology. LoC systems are a diminutive device engineered to reproduce the liver’s physiology. The main principle in vitro is to preserve continuous hepatocyte specific function. However, with advancing technology LoCs are evolving to comprise further hepatic support cells, real-time assessment of metabolites and reproduce liver zonation [30].

In vitro physiology models such as LoCs exhibit distinct advantages, as they replicate realistic physiological functions, and acquire self-organization, as the cells on the chip respond to stimuli that emulate the internal conditions of organs. Various internal and external environmental factors are required for the growth of cells. Thus, these parameters need careful consideration for the establishment of chip-based models. Beckwitt et al., 2018, found how environmental factors can be efficiently controlled through the combination of micromachining, cell biology and microfluidic technology [33]. This in turn generates mechanical stress, fluid shear stress, biochemical concentration gradient and other stimuli to mimic the liver’s internal environment.

Primary cell isolation and in vitro culture is an extensively used laboratory method. Nonetheless, many studies such as Hu et al., 2016, and Usta et al., 2015, have criticized this method for producing biased conclusions as it is far from replicating in vivo environments, as cell specific factors are often neglected [34,35]. Key cell-specific factors and microenvironment elements need to be continuously controlled. These include features such as extracellular matrix, tri-dimensional niche, chemical factors (temperature, pH, osmolarity, oxygen and carbon dioxide levels), paracrine and auto-secreted factors (growth factors, chemokines and hormones), mechanical stimuli and interaction with neighbouring cells [36]. The LoC devices overcome these limitations by replicating the organization of cells within native tissue and controlling environmental conditions for effective cell culture. 

Primary hepatocytes are often used as the ideal candidates for LoC devices as they are the most reliable cell source. However, Elaut et al., 2006, argued that they begin to desert their liver specific function within days of culture, are unable to proliferate in vitro and often de-differentiate within hours of isolation [36,37] therefore hindering their use for long-term experiments. Alternatively, Wilkening and Bader 2003 have demonstrated how many microfluidic devices have benefited from using human hepatoma cell lines such as C3A and HepG2, as they rapidly grow in vitro and are easy to handle. Unfortunately, this results in loss of liver-specific function, impeding the main mechanism of the LoC [38].

Another issue leading to controversy is the use of conventional culture configurations, such as stiff culture plates that are coated with poor extracellular matrix proteins. Some studies discussed how they fail to maintain the functional characteristics of primary liver cells in vitro. Hepatocytes begin to lose functionality and rearrange into fibroblast-like masses, due to the loss of their polygonal shape. As a result of the phenotypical and functional behaviour exhibited, softer substrates with collagen sandwich clusters, for more complex ECM compositions, and the use of decellularized scaffolds are beginning to surface. These new approaches exhibit enhanced hepatocyte features [39].

Polydimethylsiloxane (PDMS) a silicone-based organic polymer is the current biomaterial used for LoC technology, as it provides biological compatibility and efficient permeability. However, it is facing scrutiny as studies such as Wei et al., 2016 suggest PDMS absorbs small hydrophobic molecules, which results in validity issues, as experimental errors and reduction in effective concentrations are established [30]. Nonetheless, research conducted by Mauriac et al. into new polymers that exhibit the same characteristics as PDMS, such as polyurethane could be used to replace the material [40].

Lee et al., 2013 have developed a novel 3D LoC which enabled the investigation of intercellular interaction between hepatic stellate cells and spheroid hepatocytes. A distinctive feature of this LoC is that it does not require an external power source and involves minimal handling measures [41]. This is due to osmotic pumping which allows consistent flow of cells receiving specialized media, a key aspect in enabling cell growth, thus enhancing the differentiation ability of cells and their viability. It was established that efficient cell-to-cell contact is integral in cultivating liver related function and increasing the function of hepatocytes in culture [32].

The efficiency of new therapies is not being furthered through the testing of cell cultures on Petri dishes; cells exhibit different behaviour in their natural environment within the human body compared to when they are cultured. Furthermore, the use of animal studies is expensive, long and still does not provide competent evidence of effective treatment results for humans. Many drugs demonstrate validity during clinical testing on animals, however still end up attesting toxic or harmful to humans. Organ-on-a-chip system’s enable a more physiological environment by copying key factors of tissues and organs. They allow for greater control by emulating factors such as mechanical strain, shear stress and chemical gradients. It is an emerging field that is growing rapidly and offers immense potential for future operations such as toxicity testing, drug development, tissue engineering and disease modelling. The technology could allow personalized medicine by enabling individual treatment of users through the testing of their own cells.

## 3. Bioengineered Scaffolds

Liver tissue engineering (LTE) holds the potential of restoring in part or total functions of the liver, with the aim of treating acute or chronic liver diseases. The ultimate goal of LTE is to reproduce a completely functional liver to be transplanted into affected patients or be used as an extracorporeal device. Tissue engineering techniques include cell seeding or implanting cells into scaffolds with biodegradable properties and structures capable of maintaining three-dimensional (3D) cell growth. Typically, these scaffolds comprise an artificial matrix or ECM, along with a selection of polymers that offer mechanical support for 3D cell proliferation. LTE technical approaches are dependent on the use of adult hepatocytes since these cells are anchorage-dependent cells. Hepatocytes are also regarded as being very sensitive to the ECM environment for maintenance of their differentiated functions and viability. Therefore, in order to produce an effective hepatocyte cell culture, LTE requires an appropriate ECM environment [42,43].

Scaffold material selection will have a vital effect on the success of the liver tissue engineering technical approach. The scaffold should be capable of providing sufficient support for growing tissue and surface topography for successful cell attachment. Scaffolds should also be designed to provide channels for cell migration. Animal-extracted ECM scaffolds have the advantage of supplying binding sites to enable integrin-mediated cell adhesion. However, Hammond et al., 2006 discussed several problems associated to this particular type of scaffold, which include: low mechanical strength, not being immediately scaleable and experiencing interbatch variability [44]. Thus, biodegradable polymers are becoming increasingly popular within LTE. These polymers have been identified to behave more predictably in vitro, which is why they are more susceptible to being modified to improve cell-surface attachment. Additionally, they have the capability to be constructed into complex micro-scaffolds and degrade to form natural metabolites.

**Figure 3:**
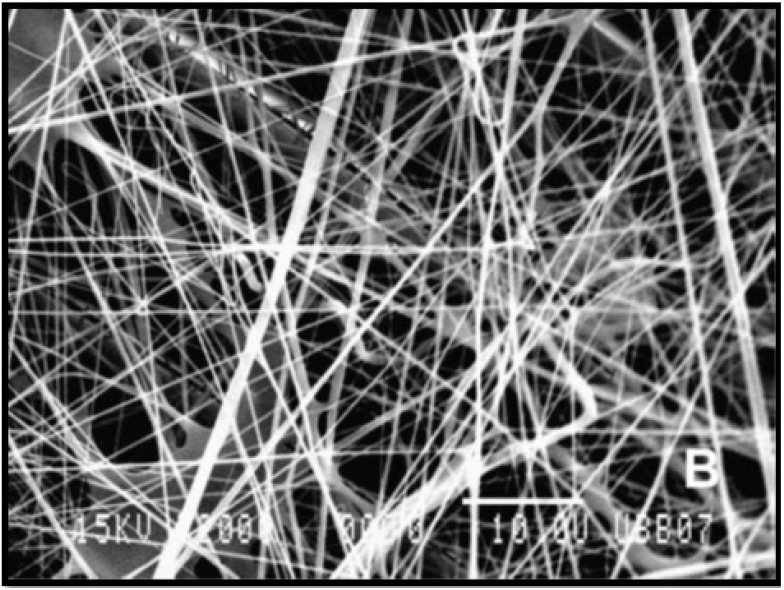
Scanning electron microscopy (SEM) micrograph (2000×) of poly (L-lactic) acid (PLLA) fibres produced from electrospinning [45].

Biodegradable polymers, which include polylactic lactic acid (PLA), poly (L-lactic) acid (PLLA) polyglycolic acid (PGA) and PDMS are frequently used to produce a scaffold (Figure 3) that provides a microenvironment comparable to the in vivo environment [31,45]. Li et al., 2013 have reported that this microenvironment includes a high supply of oxygen and nutrients, a 3D ECM, as well as high cell density. These biomaterials have been exhibited to support viable hepatocyte populations, which demonstrates their excellent biocompatibility [46]. Hammond et al., 2006, stated how various surface-modification procedures are available to increase the cell-surface adhesion rate of these biomaterials, whilst not causing any adverse effects to their bulk properties [44].

**Figure 4:**
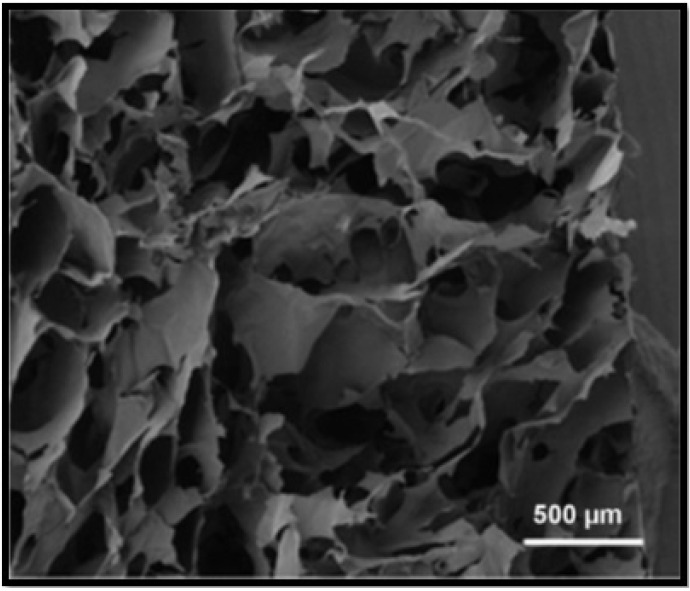
SEM of the cross–section of alginate scaffold [48].

Jain et al., 2013 have assessed different hydrophilic polymers which have been used in the production of bioengineered scaffolds for liver reconstruction [47]. Chitosan has been identified to have a likeness to glycosaminoglycan, thereby causing it to be a popular matrix for hepatocyte culture. Chitosan scaffolds manufactured as composites, nanofibers, and hydrogels are commonly used for the maintenance of hepatocytes in vitro. Being a hydrophilic polymer, chitosan has the ability to promote spheroid formation within hepatocytes. Hybrid scaffolds comprised of chitosan with collagen have been used effectively for hepatocyte differentiation and spheroid formation. Scaffolds constructed from alginate, as seen in Figure 4, have been utilised to microencapsulate or cultivate hepatocytes to develop implantable constructs [45,48]. Additionally, alginate is also a hydrophilic polymer, and as a result it stimulates spheroid formation. This consequentially increases cellular interactions, along with hepatocyte function. In order to create favourable growth conditions for hepatocyte culture, porous alginate scaffolds are produced to contain approximately 90% porosity and a pore size of 100 µm. They are capable of encouraging spheroid formation due to low cell adherence to the substrate.

Recent studies have demonstrated structure optimisation of porous and mesh scaffolds manufactured from biomaterials that retain low cell-adhesion strength. Altering the surface features of the selected biomaterial enables the possibility to further improve function of the scaffold. Hepatocytes exhibit different behaviour on a monolayer of a polymer, in contrast to a porous or mesh configuration on the same polymer. Research conducted by Edgar et al., 2016 demonstrated that porous scaffolds comprise interconnected micropores with hydrophilic characteristics and exceptional fluid absorption, resulting from their large surface area to volume ratio [49]. Moreover, their mechanically poor architecture, flexibility, and degradability, allow porous scaffolds to be a useful application for wound repair. Mesh scaffolds have become relatively popular within the TE community because of their ability to show a structural architecture similar to that of natural soft tissue. Mesh scaffolds are produced via electrospinning nanofibers that are composed of the desired biomaterial. These nanofibers are woven to form a 3D structure capable of supporting an environment for cellular adhesion and proliferation [44].

Furthermore, one of the most significant challenges to LTE is to manufacture a porous scaffold with high mechanical strength, along with having the ability to maintain vascularisation. Numerous studies emphasise that the function of the scaffold is determined by the following main factors: pore numbers, pore size, and pore connectivity. Size of pores could have a significant influence on cell migration, as extremely large pores may diminish vascularisation. In comparison, pores that are smaller than 100 nm could affect diffusion of nutrients and waste. Inefficient diffusion of nutrients could lead to decreased viability of implanted cells and overall failure of the implanted device. Therefore, the porosity must be suitably balanced with the chosen biomaterial, in addition to their mechanical features and cellular influence [50,51]. The following table (Table 1) summarizes biomaterials utilized for liver tissue engineering scaffolds.

## 4. Stem Cell Therapy

Kwak et al., 2018 conducted research on stem cell-based therapy (SCBT) used to treat liver cirrhosis and found it to have promising results in both preclinical and clinical trials. It seems SCBT may in the future become a popular alternative to orthotopic liver transplantation, which is currently the only definite treatment for a patient suffering from end-stage liver cirrhosis [58]. There is still much more to understand regarding the precise mechanisms of stem cells. Several stem cells of diverse origin have been utilised for hepatic regeneration and this section will delve further into this matter.

### 4.1. Hematopoietic Stem Cells (HSA)

Hematopoietic stem cells (HSCs) have been routinely utilised to investigate SCBT [59,60]. HSCs have a CD34 cell surface marker and are the most common type of stem cell within bone marrow [61]. HSCs can transform into progenitor cells via differentiation and can renew themselves [58,62,63]. HSC’s can also be derived from cytokine-mobilized and umbilical blood [59,60]. Blood disorders have been treated using HSCs for an extensive period, and therefore the use of the cells in SCBT is not thought to be a novel approach [60,64].

Jang et al., 2004 carried out a study to see how in vivo/in vitro HSCs behaved towards injury of the liver, since functional and phenotypic changes occur within the liver in this instance. It was found that HSCs have the potential to differentiate into liver cells when they are co-cultured with an injured liver [65]. The in vitro experiment displayed that microenvironmental cues had a hand in the transformation of HSCs into liver cells. This was found through analysis of protein expression, chromosomal studies and tissue-specific genes. A trend was noticed when HSCs were transplanted into mice who had liver injuries, it was noticed that viable hepatocytes were being introduced as the injury progressed. The restoration of liver function occurred between two to seven days after the HSC transplantation occurred. From research it can be concluded that HSCs can differentiate into functional hepatocytes and contribute towards the regeneration of an injured liver. Lagasse et al., 2000 experimental results further support this conclusion. Adult bone marrow cells were intravenously injected into a tyrosinemia type I affected FAH mouse and ultimately only HSC cells gave rise to hepatic regeneration [66].

From the research of Liu et al., 2006, liver regeneration (after partial orthotopic liver transplant) can be induced by mobilizing HSCs with granulocyte-colony stimulating factor (G-CSF). It is less difficult to conduct this treatment in a universal way. Current therapeutic procedures such as this one, have a basic concept of mobilizing, proliferating and directing stem cells to improve recovery from liver injury by a partial liver transplantation. Although stem cells can be linked to restoring liver function, it is not yet possible to define their exact action in the liver regeneration process. Tsolaki et al., 2014 reiterated this uncertainty since they also were unsure if the liver regeneration processes were triggered or mediated by the on-site HSCs and/or by the effect of the G-CSF. This further emphasises the difficulty of characterising exact root causes for hepatic regeneration. Additionally, G-CSF alongside other mobilization agents such as Plerixafor and a G-CSF+Plerixafor combination can potentially aid the reversal of chronic liver injury via unique processes. A clear observation was made that the G-CSF possessed the greatest anti-fibrotic ability when it was used for an extended period. This result further proves the link between G-CSF and its anti-fibrotic outcome [67].

### 4.2. Mesenchymal Stem Cells (MSC)

Mesenchymal stem cells (MSCs) are being thoroughly investigated for the purposes of regenerative medicine as they possess distinct biological properties [68]. MSCs can be extracted from adipose tissue, umbilical cord blood and bone marrow. Since MSCs can undergo extensive expansion within in vitro conditions, this allows them to swiftly reach the desired quantity when required for in vivo therapy [69,70]. Mesenchymal stem cell (MSC) can be differentiated into hepatocytes, promote liver regeneration, inhibit liver fibrosis and induce liver apoptosis, particularly via paracrine mechanisms [71]. Dominici et al., 2006 states that it is difficult to compare results from research papers investigating MSCs due to the different procedures used for isolation, expansion and characterization of cells, which in turn hinders progress in this field of study [72]. To combat this issue, the International Society for Cellular Therapy has defined a criteria list to characterize the human MSC. Firstly, it is crucial that the MSC displays plastic adherence when stored in a standard cell culture environment. Additionally, the MSC must express (CD90, CD105, CD73) and lack expression of (HLA-DR, CD34, CD14 or CD11b, CD79alpha or CD19 and CD45) certain cell surface molecules. Finally, the MSC must have the differential ability to transform into adipocytes, chondroblasts and osteoblasts in vitro.

Though isolated MSCs all express the MSC marker profile, they are commonly diverse in both differentiation and proliferation. In vitro cultivated MSCs have excellent differentiation ability, immunoregulatory potential and can aid tissue remodelling through the emission of trophic factors. These key properties make MSCs useful in cellular therapy [73,74]. The review paper by Shiota and Itaba 2016 found that MSCs have the potential to improve liver diseases such as fatty liver disease, acute liver failure and liver fibrosis. MSCs have great versatility and, therefore, they can differentiate into hepatic lineages. In one study, HNF3β was expressed when a tetracycline-controlled system was regulated in the UE7T-13 (bone marrow derived) MSCs. This expression caused a ripple effect, since it also increased the expression of alpha-fetoprotein, epithelial cell adhesion molecules, albumin and tyrosine amino transferase genes. When tetracycline treatment was continued over 8 days, it was seen that majority of the MSCs expressed albumin. Albumin is linked to the maturing of hepatocytes. It can be concluded that MSCs are a valuable source to use in SCBT to treat liver disease [70,75].

Gao et al., 2017 carried out an investigation with (adipose-derived) MSCs and found they are useful for suppressing rejection in reduced sized liver allografts. Regulatory T cell production is stimulated by MSCs; this is key in its immunosuppressive effects. MSCs were also found to boost hepatocyte regeneration and hinder hepatocyte apoptosis, which in turn promotes liver regeneration [76]. Liu et al., 2018 explored (bone marrow-derived) MSCs and found evidence that MSC transplantation could potentially aid recovery for patients who have undergone a liver resection. Liver regeneration occurs since MSCs encourage cell proliferation/growth and MSC infusion can potentially improve lipid accumulation within the liver [77].

### 4.3. Embryonic Stem Cells (ESC)

**Figure 5:**
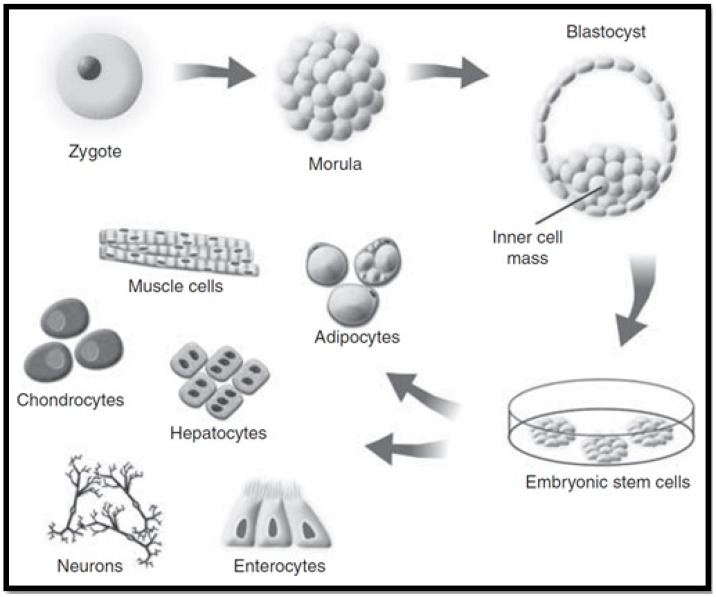
Embryonic stem cell cultivation [78].

Embryonic stem cells (ESCs) are categorised as pluripotent stem cells that are extracted from the inner cell mass of blastocyst stage embryos. These stem cells retain the potential to self-renew indefinitely, whilst having the ability to differentiate into all cell types in the human body (as seen in Figure 5) when supplied with the relevant stimulus for differentiation [48,78]. This high degree of differentiation and unlimited potential for self-renewal, allow ESCs to be considered as an effective regenerative therapy for tissue replacement following liver disease. Tsolaki and Yannaki, 2015 discussed that ESCs have the capacity to proficiently differentiate into hepatocyte-like cells in vitro, creating cells that have some similar properties to that of mature hepatocytes [79]. For this reason, ESCs are a valuable option for studying the molecular source of hepatocyte differentiation. A recent study by Tolosa et al., 2015 identified an effective method for the differentiation of ESCs into neonatal hepatocytes that had the ability to reintroduce livers in vivo without instigating tumour development in mice [80].

However, there are some limitations to the use of ESCs in cellular therapies. The fact that the derivation of ESCs requires the destruction of blastocyst stage embryos raises ethical issues that have slowed the advancement of ESC research. Tsolaki and Yannaki, 2015 emphasise that regardless of the significant progress and development of complex differentiation methods mimicking embryonic development, ESCs extracted from hepatocyte-like cells are unable to completely function as mature adult hepatocytes [79]. Furthermore, the concern of immunological incompatibility of the transplanted cells, hinder their application as a cell replacement therapy [81].

### 4.4. Induced Pluripotent Stem Cells (iPSC)

**Figure 6:**
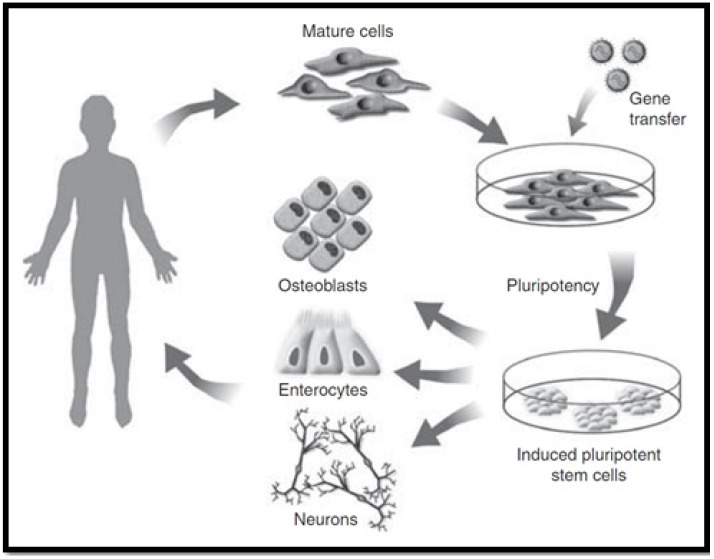
Induced pluripotent stem cell cultivation [78].

Induced pluripotent stem cells (iPSCs) have properties comparable to those of ESCs, such as self-renewal and pluripotency, while avoiding the main issues that are associated with the use of ESCs. iPSCs can be reprogrammed from somatic cells through the use of pluripotency factors. Forbes et al., 2015 established that they do not require embryonic material (as seen in Figure 6) [78], thereby obviating ethical controversies [82]. In addition, iPSCs hold the potential to be clinically beneficial since they can be formed from autologous stem cells, which provides the opportunity for autologous use and avoiding the condition for immunosuppression [83]. In the event that iPSCs are used for the derivation of hepatocyte-like cells for treatment of a genetic liver disorder, then the autologous source would need some type of “gene surgery” before use. Nicolas et al., 2016 stated that the use of iPSCs as a cellular therapy date back to 2006, and has drastically grown in popularity as a favourable alternative to ESCs [81]. However, before considering their clinical applications, several issues need to be resolved.

iPSCs have increased expectations for regenerative medicine due to their potential to deliver personalised treatment, while their derivation from patient-specific cells overcomes the risk of rejection. Although iPSCs are an interesting alternative to ESCs, there are some issues that need to be addressed before this new form of cellular therapy can move from proof of concept to the clinic. Yu et al., 2014 suggests that an in-depth preclinical evaluation of iPSCs in appropriate large animal models is essential to make sure that treatment with iPSC-derived cells is safe and efficacious before human trials [84]. Hence, important concerns need to be addressed, which include effectiveness of the iPSC-based treatments, and long-lasting safety tolerability. It is vital that the optimal reprograming method is established by using clinically applicable standardised protocols. Cost-efficient manufacturing techniques need to also ensure that they can support the development of rapid differentiation methodologies for culturing mature cell types from iPSCs. Despite these restrictions, iPSC-derived hepatocytes still promise innovative solutions for liver cell therapy, and donor grafts extracted from iPSCs could potentially provide suitable organs for liver transplants [79].

### 4.5. Endothelial Progenitor Cells (EPCs)

Endothelial progenitor cells are categorised as immature endothelial cells that are located within bone marrow and peripheral blood vessels. These cells manifest from hemangioblasts and are responsible for the neovascularisation of damaged tissue in the body. However, Kwak et al., 2018 emphasises that EPCs are likely to be derived from several cell lineages, which is proven by their various surface markers [58]. According to an animal study conducted by Nakumura et al., 2007, transplantation of EPCs resulted in the intervention of liver fibrosis by successfully suppressing the activation of HSCs. These EPCs were also exhibited to stimulate hepatocyte proliferation and greater matrix metalloproteinase activity, in addition to increased secretion of growth factors [85]. Rautou, 2012 states that, in vitro, EPCs are identified by their capability to produce adherent cell populations that differentiate and multiply into endothelial lineage. When in vivo, EPCs contribute to the dynamic process of angiogenesis in ischemic sites or assist with vascular repair following damage to vessel walls [86]. Accumulating data display that these characteristics of EPCs detect a heterogeneous cluster of cells with respect to their origin, differentiation and functional properties. 

SCBT has been deemed an alternative treatment for liver diseases since promising results have been found via clinical and experimental research. A range of stem cells (including HSCs, MSCs, iPSCs, EPCs, ESCs) have been explored to investigate their clinical potential and viability. Extensive research has been carried out with MSCs, which results in a broader understanding of the potential regenerative liver treatments available. Long-term efficacy is still unproven and experimental trials do not have standardized protocols, which is disadvantageous. However innovative technologies of the future are likely to resolve the current issues associated with SCBT [58]. Table 2 summarizes the derivation of stem cells that have been derived from their respective locations and additional information.

## 5. Bioartificial Livers

**Figure 7:**
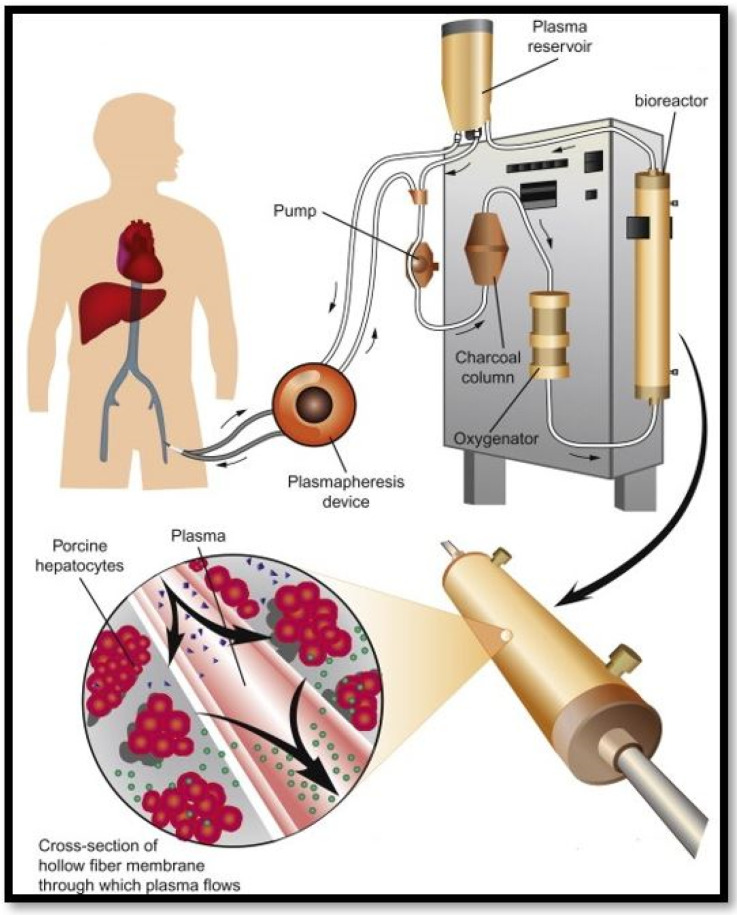
Bioartificial liver support system [91].

Liver failure is a rising problem resulting in many deaths worldwide. Only 10% of individuals in the UK who require a liver transplant obtain one [87]. Therefore, alternative treatment options are vital. As an alternative therapy, artificial liver support systems may bridge patients to liver transplantation or spontaneous recovery [88]. A bioartificial liver (BAL) machine could provide transient liver function, enabling time for the patient’s liver to repair and regenerate or at least buy time until a suitable donor organ can be allocated [89]. Bioartificial livers make use of viable hepatocytes to adopt liver specific functions. Most BAL systems (as seen in Figure 7) utilize a hollow fibre technology and are reinforced by a bioreactor where they are cultured and stimulated to process blood of liver failure patients [90]. They provide plasma detoxification through absorption procedures and dialysis filtration.

Over the past decade BALs have been presumed to be a favourable treatment for liver failure. Nonetheless, the clinical application of these support systems is still in the developmental process. Numerous studies such as Stutchfield et al., 2011 and Sakiyama et al., 2017 have analysed the development of extracorporeal bioartificial livers, however, the effectiveness of BALs has not been documented [92,93]. Furthermore, a consensus regarding the features that these systems should possess to efficiently adopt liver functions in individuals is still being deliberated. Recentl research into in vitro BAL applications such as disease modelling and drug development has identified subsiding clinically relevant characteristics [94,95].

The advancement of BAL systems recently as a tissue engineered extracorporeal device has been substantial. However, the clinical application of these devices is still debatable, due to several features that have not been adequately addressed. Starokozhko and Groothuis 2017 considered how one of the main limitations of BAL’s is the absence of a suitable cell source. Previously used cell sources for BAL treatment in large animal models and patients consist of primary human hepatocytes, primary porcine hepatocytes and human liver tumour derived cell lines. The most common biological component utilized in BALs are primary porcine hepatocytes. There is an immense degree of metabolic resemblance between pig and human hepatocytes, however, they are repeatedly observed as being unable to manufacture coagulation factors and often lead to zoonoses and immunogenicity restrictions [94].

Primary human hepatocytes are perceived to be a more suitable alternative; however, many studies such as Zhao et al., 2012 demonstrated the limitations represented by this cell source especially regarding the vacant quality and yield. They conversed how the availability of adequate donor livers far surpasses the demand thus, accessibility for BALs is limited to discarded organs and tissues that often entail steatosis and fibrosis [96]. Furthermore, Elaut et al., 2006, argued that primary human hepatocytes often de-differentiate within hours of isolation, begin to desert their liver specific function within days of culture, and are unable to proliferate adequately in vitro. Thus hindering their use for long term experiments [37].

The clinical application of BAL support systems can only be furthered when the biological component of an adequate cell source is obtained. The main aim for future studies in order to develop an appropriate cell source should be to determine a mature human hepatocyte with proliferative capacity in vitro, and characteristics of an unlimited life span with a diminished probability of zoonosis transmission and immunogenicity [97]. Regrettably, a cell source with these characteristics is yet to be discovered. A highly deferential human hepatocyte line was the most ideal candidate; nevertheless, studies such as Lee et al., 2015, have claimed liver functions cannot be substituted with just a single cell line [98]. The liver is composed of a variety of cell types such as Kupffer cells, hepatocytes and sinusoidal endothelial cells that maintain the physiology of the liver through communication. Therefore, coculture techniques should be pondered for acquiring a suitable cell source for bioartificial systems in vitro.

Another disadvantage in the design of the existing BAL bioreactor is the inability of the system to perform bile secretory function, which is classed as one of the main roles of the liver. BAL devices do not have a system in place that moves the bile produced by hepatocytes out of extracorporeal circulation. Instead, as noted by Zhang et al., 2018, a combination of an artificial liver device and a BAL bioreactor, such as plasma exchange, or albumin dialysis have been adapted. However, devices such as these are debatable, as evidence regarding whether the artificial liver device can remove the bile produced is questionable, and the percentage of bile retained intracellularly is unclear. Thus, the efficacy and clinical efficiency are difficult to discern [96,99].

Bioartificial support systems are continuing to evolve and are perceived to be promising candidates to overcome the prevailing demand for liver transplantation. Nonetheless, the insufficient clinical evidence and the availability of the quality and yield of an adequate cell source, restrict the possibilities of a system that can offer immense potential. Extracorporeal BAL systems provide patients with a chance to promote liver regeneration, or at least assist them until a suitable donor is located for liver transplantation. Further research regarding this life-saving technology should focus on developing a method to produce in bulk a high-quality, readily available, differentiated human hepatocyte line. Correspondingly, a greater understanding concerning the formation of liver microstructure and hepatocyte proliferation should be deliberated for widespread clinical application of BALs [96,98,100]. Figure 3 summarises the pros and cons of the regenerative treatment options available for patients affected by liver disease.

Finally, it is worth mentioning the latest technique including 3D printing and bioprinting which are under investigation by many research institutes to tackle issues related to liver diseases. Three-dimensional (3D) printing or rapid prototyping is a well known technique and has become very popular for the past 10 years in the field of tissue engineering and regenerative medicine. These techniques will assist in the generation of 3D objects through the processing of computer images [101]. Three-dimensional printing has various applications in liver transplantation including surgery. Due to the complexity of liver function and its anatomy, it is not an easy task to fully generate a liver organ without considering all parameters within the human body and, for this reason, at least 5 to 10 years investigation is required to print a liver with at least 50% of its original functionality.

## 6. Conclusions

The main effective regenerative therapy for progressive liver diseases is liver transplantation. The high demand for compatible donor livers surpasses the availability. The rising age of the population with modern lifestyles has increased the number of individuals suffering from liver disease. This has led to extensive research into regenerative treatment options such as the tissue engineering possibilities discussed within this paper. A promising candidate to overcome this demand is SCBT. In particular, iPSC-derived hepatocytes offer innovative solutions for liver stem cell-based therapy. iPSCs hold the potential to be clinically beneficial since they can be formed from autologous stem cells, and this provides the opportunity for autologous use thereby avoiding immunological incompatibility. Also, donor grafts extracted from iPSCs could potentially provide suitable organs for liver transplants whilst obviating ethical issues regarding destruction of blastocyst stage embryos.

LoC systems are another emerging alternative treatment option. They allow more efficient control by mimicking the physiological conditions of the liver such as shear stress, chemical gradients, and mechanical strain. It is an advancing field of technology that provides endless possibilities within the tissue engineering sector. Microfluidic chip-based technology may one day allow for personalized medicine, thus reducing the probability of immunological rejection. Furthermore, human-scale bioartificial devices are continuing to evolve however, due to the inadequate clinical evidence the efficacy of these is still questionable. Nonetheless, LoCs may be able to further the progression of effective BAL systems through the experimental analysis of potential cell sources and patient-specific biomaterials.

Overall, SCBT experimental protocols must be standardized in order to efficiently compare results across various studies and allow progression within this field. The long-term efficacy of SCBT is still unproven; therefore, further experimental work must be conducted, and this will improve understanding of the benefits and drawbacks. The lack of clinical evidence for regenerative treatment is one of the major limitations of LTE therapies. For instance, large animal models could be used to ensure that treatment with iPSC-derived cells is safe and efficacious before human trials.

## Figures and Tables

**Table 1:** Summary of biomaterials used for scaffolds in liver tissue engineering.

Biomaterials Utilised for Liver Tissue Engineering Scaffolds			
Biomaterial	Advantages	Disadvantages	Ref
Polylactic Acid (PLA)	Biocompatible Nanofibers can mimic native extracellular matrix architecture Low cost Physical properties can be tailored via different scaffold preparation method Good mechanical properties	Without surface modification poor cell adhesion occurs Possible undesired inflammatory responses and toxicity Hydrophobicity can hinder successful cell seeding	[46,52,53,54]
Poly (L-lactide) (PLLA)	Isomer form of PLA Biocompatible Can be fabricated to desired architecture Degradation characteristics alter by changing composition	Without surface modification poor cell adhesion occurs Degradation can lead to cell/tissue necrosis	[41,55]
Polyglycolide (PGA)	Biocompatible Can be fabricated to desired architecture Degradation characteristics alter by changing composition Good mechanical properties	Without surface modification poor cell adhesion occurs Possible undesired inflammatory responses and toxicity Degradation can lead to cell/tissue necrosis Hydrophobicity may hinder successful cell seeding	[41,52,53,55]
Polydimethylsiloxane (PDMS)	Biocompatible Efficient permeability Biodegradable	Absorbs small hydrophobic molecules	[30,40,41]
Chitosan	Hydrophilic polymer Promotes spheroid formation within hepatocytes Increases cellular interactions Increases hepatocyte function Good mechanical strength Biodegradable	Poor mechanical properties in comparison to synthetic polymers Membranes are very stiff and brittle, resulting in low mechanical resistance	[47,52,55,56]
Alginate	Hydrophilic polymer Promotes spheroid formation within hepatocytes Increases cellular interactions Increases hepatocyte function Biodegradable	Poor mechanical properties in comparison to synthetic polymers Very limited mechanical stiffness	[47,55,57]
Animal extracted ECM	Supplies binding sites to enable integrin-mediated cell adhesion	Low mechanical strength Not immediately scaleable Interbatch variability	[44]

**Table 2:** Summary of derivations and key facts for; stem cells including hematopoietic stem cell therapy, mesenchymal stem cells, embryonic stem cells, induced pluripotent stem cells and endothelial progenitor cells.

Derivation of Stem of Stem Cells		
Stem Cell Type	Derivation	Key Facts
**HSC**	Cytokine mobilised blood Umbilical blood	Can transform into progenitor cells via differentiation Self-renewal potential Can undergo cell apoptosis Difficult to identify as their morphology is similar to white blood cells in culture
**MSC**	Adipose tissue Umbilical cord blood Bone marrow Fallopian tube Foetal liver and lung	Multipotent stromal cells Differentiation potential High immunoregulatory potential Crucial for repairing cartilage, bone and skeletal tissue
**ESC**	Blastocyst Stage Embryos	High degree differentiation Unlimited potential for self-renewal Greatly versatile and durable Can be used in testing drug safety and effectiveness
**iPSC**	Somatic cells	Can be reprogrammed from somatic cells through use of pluripotency factors Avoids destruction of embryos High pluripotency Can be used for personalised treatment as they are derived from autologous cells Avoid immunological incompatibility
**EPC**	Bone marrow Peripheral blood vessels	Responsible for neovascularization of damaged tissue Potential to hinder liver fibrosis by suppressing activation of HSCs Stimulate hepatocyte proliferation and greater matrix metalloproteinase activity Increase secretion of growth factors

## Data Availability

Not applicable.
